# Diagnostic accuracy of early warning system scores in the prehospital setting

**DOI:** 10.12968/jpar.2023.15.12.516

**Published:** 2023-12-02

**Authors:** S Bell, JE Hill

**Affiliations:** North West Ambulance Service NHS Trust; University of Central Lancashire

## Abstract

The utilisation of pre-hospital early warning scores in ambulance services is widely endorsed to promptly identify patients at risk of clinical deterioration. Early warning scores enable clinicians to estimate risk based on clinical observations and vital signs, with higher scores indicating an elevated risk of adverse outcomes. Local healthcare systems establish threshold values for these scores to guide clinical decision-making, triage, and response, necessitating a careful balance between identifying critically unwell patients and managing the challenge of prioritisation. Given the limited evidence for optimal early warning scores in emergency department and pre-hospital care settings, a systematic review by Guan et al. (2022) was undertaken to assess the diagnostic accuracy of early warning scores for predicting in-hospital deterioration when applied in the emergency department or pre-hospital setting. This commentary aims to critically appraise the methods used within the review Guan et al (2022) and expand upon the findings in the context of clinical practice.

## Introduction

The use of pre-hospital early warning scores (EWS) in ambulance services settings is widely advocated, with their using seeking to identify early in their clinical course patients at risk of clinical deterioration (1). EWS allow the clinician to calculate a risk score for an individual patient (2). This score is based upon their clinical observations and vital signs at the time of assessment with the resulting score providing indication as to risk (3). Higher scores are indicative of a higher risk of adverse outcome and deterioration and serve to identify patients requiring an increased clinical response (4). EWS can be applied across a range of conditions and may be generic in nature, although specific tools also exist for specific conditions such as sepsis (5). Local healthcare systems set threshold values for the resultant score to guide clinical decision-making, triage, and response decisions (6). Care must be taken to maintain a balance, ensuring that the risks of overlooking potentially critically unwell patients are weighed against the challenge of prioritising too many patients and overwhelming healthcare systems (6). Acknowledging that compared to in-hospital ward settings, there is little published evidence to determine the optimal EWS for emergency department and pre-hospital care use, the systematic review undertaken by Guan et al (2022) seeks to determine which EWS best predicts in-hospital deterioration of patients when applied in the Emergency Department (ED) or within the pre-hospital setting (7). This systematic review and meta-analysis aimed to articulate the pooled odds of predicting clinical deterioration in hospitalised patients through the stratification of the EWS score as determined in the ED and pre-hospital settings. The impacts assessed included short (≤3-day) and long-term (≤30-day), mortality and ICU admission, together with overall lengths of hospital stay and cardiac or respiratory arrests all investigated through consideration of the current evidence base.

### Aim of commentary

This commentary aims to critically appraise the methods used within the review Guan et al (2022) and expand upon the findings in the context of clinical practice.

### Methods

This pre-registered systematic review undertook a comprehensive multi-database search from date of inception to February 2021. Screening of all included studies were undertaken to identify additional papers. Only experimental, quasi-experimental, or observational studies published in English which assessed EWS in individuals aged 14 or older in either an emergency department or pre-hospital settings were included. The five tests of focus were; Cardiac Arrest Risk Triage (CART), Rapid Acute Physiological Score (RAPS), Modified Early Warning Score (MEWS), National Early Warning Score 1 & 2 (NEWS 1 & 2). These tests were assessed regarding their ability to predict both short-term mortality (3 days) and long-term mortality (30 days). Screening, data extraction and assessment of quality (Newcastle-Ottawa Scale) was undertaken by at least two reviewers independently. A meta-analysis was conducted utilising a random-effects model to calculate a diagnostic odds ratio (DOR) along with its corresponding 95% confidence interval. Heterogeneity was assessed using the I^2^ statistic. Publication bias was assessed by visual inspection of a funnel plot. A sensitivity analysis was conducted to evaluate the impact of the high risk of bias studies.

### Results

After duplicate removal, 8972 papers were identified of which after screening 20 papers were included within the review. Among these included studies, only seven papers were conducted in the pre-hospital setting. The remainder of the studies were carried out within emergency departments. Two studies were classified to be of poor quality; in a sensitivity analysis, when these two studies were excluded, it was observed that their removal did not yield a significant impact on any of the results.

When evaluated for diagnostic accuracy in predicting up to 3-day mortality within the **pre-hospital setting**, it was noted that NEWS2 predictive score cut-off points of both ≥5 (DOR 14.06, 95% CI: 9.09 to 21.75, I^2^ = 0%,) and ≥7 (DOR 12.26, 95% CI: 8.58 to 17.64, I^2^ = 4.4%) generated comparable DORs. At a threshold of ≥9, there was a notable enhancement in DORs (DOR 20.37, 95% CI: 13.16 to 31.52, I^2^ = 0%). However, owing to substantial imprecision in the estimates observed across all three analyses, the difference between the three thresholds did not achieve statistical significance. Similarly, the NEWS demonstrated a comparable level of accuracy to NEWS2 when both were evaluated at the same cut-off threshold of ≥7 (DOR 11.63, 95% CI: 9.75 to 13.88, I^2^ = 0%) within the pre-hospital setting. When evaluated for predicting up to 30-day mortality, a NEWS threshold of ≥7 demonstrated a relatively low diagnostic accuracy within the pre-hospital setting (DOR 2.58, 95% CI: 0.59 to 11.21, I^2^ = 99.5%).

When evaluated for diagnostic accuracy in predicting up to 30-day mortality within the **emergency department** there was no statistically significant difference of diagnostic accuracy between MEWS ≥3 (DOR 4.05, 95% CI: 2.35 to 6.99, I^2^ = 73.0%), ≥4 (DOR 6.48, 95% CI: 1.83 to 22.89, I^2^ = 90%) and NEWS ≥6 (DOR 4.92, 95% CI 2.71–8.96, I^2^ = 65.5%). Similarly, there was no statistically significant difference of diagnostic accuracy in predicting up to 30-day mortality within sepsis patients within emergency departments between MEWS ≥5 (DOR 3.05, 95% CI: 2.00 to 4.65, I^2^ = 0%) and NEWS ≥7 (DOR 4.74, 95% CI: 4.08 to 5.50, I^2^ = 0.0%). The diagnostic accuracy for MEWS ≥3 for predicting ICU admission was DOR 5.54 (95% CI: 2.02 to 15.21, I^2^ = 50.9%). A meta-regression was undertaken for diagnostic accuracy in predicting up to 30-day mortality within emergency departments. Unfortunately, it is not indicated which tool this assessment was undertaken on and at which threshold. Although it was indicated that 92% of the variance within whatever threshold was assessed could be explained by variation in age. An Additional funnel plot assessment of publication bias using Deeks’ funnel asymmetry tests was undertaken but was none significant at the highest and lowest thresholds.

### Commentary

Critical appraisal of the authors’ methods applied in undertaking the review, assessed against a Joanna Briggs Institute (JBI) Critical Appraisal Tool for Systematic Reviews (8) reveals a high methodological standard with all criteria achieved, demonstrating a robust process (11 out 11). The completeness and high-quality approach to the methodology instils confidence that this review provides a comprehensive summary, and contextualisation of the published evidence on the topic. While the methodological approach to this review was sound, the pre-hospital clinician should read and interpret the results with an awareness of the limitations identified by the authors. These limitations include the lack of power to evaluate medical versus trauma conditions, the limited availability of data pertaining to cardiac and/or respiratory arrest outcome, and the possibility of unknown confounders impacting hospital stay. This, together with knowledge that only seven papers of the twenty papers included in the review were from studies conducted in either the pre-hospital setting or utilising pre-hospital data, should inform the interpretation of the review’s findings and their translation to pre-hospital or paramedic practice.

The review demonstrated that the cut-off points applied to EWS within the emergency department setting are lower than those used in the pre-hospital setting, within the studies included for predicting thresholds. The reporting of high cut-off points in the pre-hospital setting is potentially due to the need to strike a balance in sensitivity and specificity since lower cut-off points would theoretically result in poorer sensitivity in the pre-hospital setting. This is compounded by the short duration of the interaction between pre-hospital clinicians and patients potentially affecting the ability to achieve a reliable EWS.

From a pre-hospital perspective, the findings of the review conducted by Guan and colleagues suggest that EWS scores applied in the pre-hospital setting may not accurately predict long-term events of 30-day mortality. This is potentially of relevance to the pre-hospital clinician in the context of the observation that EWS in the pre-hospital setting appear to be more accurate when managing more critically ill or compromised patients and may not therefore be as applicable to patients outside of this cohort. As the balance between urgent and emergency presentations to ambulance services shifts towards those with urgent rather than emergency care needs, it may be the case that there is less reliability of EWS for those who potentially make up a large proportion of the population served by ambulance clinicians (9). However, caution must be applied to this inference given the large range in the confidence intervals presented and the non-statistically significant findings, and substantial heterogeneity found. Given these issues there is a significant degree of uncertainty in this result and the ability to draw definitive conclusions from the evidence presented within the review. In a more specific systematic review looking at only NEWS and NEWS2 in any clinical setting found similar findings regarding these tools having poor predictive accuracy for all deaths within 30 days (10).

The review did however demonstrate that EWS scores used in the pre-hospital setting can predict short term clinical decline (up to 3-day mortality). With NEWS2 is now widely adopted across ambulance services in England, it is important to be aware of the varying diagnostic accuracy produced at different thresholds (11). When comparing different threshold scores of NEWS2, there was no distinct differentiation in the test’s ability to predict up to 3-day mortality. This limited differentiation between tests was mainly caused by the wide confidence intervals presented. Although the review findings suggested that a NEWS2 score ≥9 might offer improved diagnostic accuracy, yet this finding lacked statistical significance when compared to alternative thresholds and tests. Pre-hospital clinicians should take note that the observations about the wide range of confidence intervals in the review’s results still hold true, although to a lesser extent than in the case of long-term events. This variance in confidence intervals reduces the certainty of the presented estimates.

These findings related to NEWS2 are in harmony with a recent, slightly broader systematic review that delved into the diagnostic accuracy of short-term mortality prediction using EWS in the outpatient emergency care scenario (12). This review used a slightly different method of assessment regarding a descriptive analysis of the area under the receiver operating characteristic curve. Unfortunately, as with the diagnostic odds ratio doesn’t give you additional information regarding specificity and sensitivity as it’s a combination of both which make up this estimate. Nevertheless, it does align with the findings that NEWS2 is reasonably accurate in predicting short-term mortality.

As highlighted in this review there is still substantial uncertainty regards to the predictive ability of EWS tools within the pre-hospital setting. Within emergency department setting, the meta-regression highlighted it is possible that the moderating factor of age may influence these tools’ ability to predict short-term and long-term mortality. However due to the limited number of studies within the pre-hospital setting, this valuable analysis was unable to take place. Therefore, future studies should aim to report and explore moderating factors in the long-term predictive ability of these tools within the pre-hospital setting, together with aiming to reassess the tools identified in this review aiming to assess similar thresholds.

In evaluating long-term predictive capabilities in the pre-hospital setting, only the older NEWS tool was able to be assessed, highlighting the need for future research to scrutinise the newer NEWS2 for its long-term diagnostic predictive accuracy. Additionally, this review exclusively presented a combined measure of diagnostic odds ratio, lacking the exploration of how the tool performs in terms of sensitivity and specificity. Therefore, future reviews should not only assess diagnostic odds ratio, but also report both sensitivity and specificity along with subsequent measurements to provide a comprehensive understanding of the tool’s diagnostic performance.

This review found that the application and study of EWS scores within the emergency department is well documented, but only limited studies and evidence was found to assess their applicability in the pre-hospital setting. This finding, together with the results of systematic review and particularly meta-analysis, entail a degree of caution is necessary in drawing definitive conclusions regarding the use and reliability of EWS in the pre-hospital context. Whilst future research may lead to further improvements and refinements to EWS for the identification of risk of deterioration in patients presenting in the pre-hospital context, scores based on currently measured physiological parameters will need careful consideration regarding sensitivity and specificity to ensure clinical cut-offs and decision making deliver real improvements over the current available EWS.

### CPD reflective questions

1. What factors should be considered when interpreting the results of this review?
2. If you use a EWS tool in practice what score/threshold do you use and why?
3. Within your own clinical practice what issues do you find when using a EWS tool and is there anything you can do to reduce these factors?

This research was partly-funded by the National Institute for Health and Care Research Applied Research Collaboration North West Coast (NIHR ARC NWC). The views expressed are those of the authors and not necessarily those of the NHS, the NIHR, or the Department of Health and Social Care.

## Key Points

- NEWS2 may provide reasonable predictive diagnostic accuracy at threshold of ≥5, ≥7 and ≥9 for predicting up to 3-day mortality within the acute hospital setting when calculated in the pre-hospital phase‥
- NEWS and NEWS2 produced similar predictive diagnostic accuracy at a threshold of ≥7 for predicting up to 3-day mortality within the acute hospital setting when calculated in the pre-hospital phase.
- There is limited, inconsistent and inconclusive evidence that NEWS2 at a threshold of ≥7 can reliably predict up to 30-day mortality within the acute hospital setting when calculated in the pre-hospital phase.
